# Supplementary material for: Causal Relationship between Aging and Anorexia Nervosa: A White-Matter-Microstructure-Mediated Mendelian Randomization Analysis
Source: Biomedicines. 2024 Aug 16;12(8):1874. doi: 10.3390/biomedicines12081874 (PMC11351342; doi:10.3390/biomedicines12081874)
Supplement: Supplementary file 1 [file biomedicines-12-01874-s001.zip › Supplementary Figure/Figure S8/Leaveoneout plot of MR analysis for TL to ubm-b-2009.pdf]

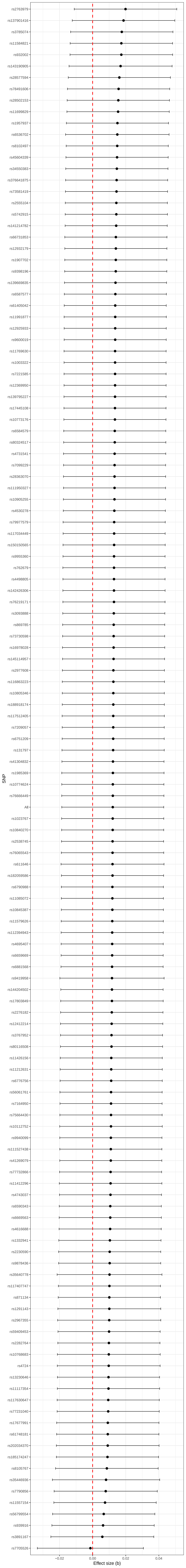
rs2763979
rs137901416
rs3785074
rs11584821
rs932002
rs143190905
rs28577594
rs78491606
rs28502153
rs11699829
rs1957937
rs6536702
rs8102497
rs45604339
rs34550383
rs376641875
rs73581419
rs2555104
rs5742915
rs141214782
rs66731853
rs12932179
rs1907702
rs9398196
rs139669835
rs6587577
rs61405042
rs11991877
rs12925933
rs9600019
rs11769630
rs1003322
rs7221585
rs12369950
rs139795227
rs17445108
rs10773176
rs6584579
rs80324517
rs4731541
rs7099229
rs28363070
rs111950327
rs10905255
rs4530278
rs79977579
rs117034449
rs150150565
rs9955360
rs762679
rs4498805
rs142426306
rs76219171
rs3093888
rs869785
rs73730598
rs16978028
rs145114957
rs2977608
rs116863223
rs10805346
rs188918174
rs117512405
rs7209057
rs6751209
rs131797
rs41304832
rs1985369
rs10774624
rs76666449
All
rs1023767
rs10840270
rs2538745
rs76065543
rs611646
rs182059586
rs6790988
rs11085072
rs10845387
rs11579626
rs112394943
rs4695407
rs6659669
rs6881568
rs9419958
rs144204502
rs17803849
rs2276182
rs12412214
rs3767952
rs80116508
rs11426156
rs11212631
rs6776756
rs56061761
rs7164950
rs75664430
rs10112752
rs9940099
rs111527438
rs41269079
rs77732866
rs11412296
rs4743037
rs6590343
rs6669563
rs4616688
rs1332941
rs2230590
rs9878436
rs35640778
rs117407747
rs871134
rs1291143
rs2967355
rs59409453
rs2282764
rs10768683
rs4724
rs13230646
rs11117354
rs117630647
rs77231040
rs17677991
rs61748181
rs202034370
rs185174247
rs8105767
rs35446936
rs7790856
rs11557154
rs56799554
rs939916
rs3891167
rs7705526
SNP
−0.02
0.00
0.02
0.04
Effect size (b)
